# *TFRC* upregulation promotes ferroptosis in CVB3 infection via nucleus recruitment of Sp1

**DOI:** 10.1038/s41419-022-05027-w

**Published:** 2022-07-11

**Authors:** Lu Yi, Yanan Hu, Zhixiang Wu, Ying Li, Min Kong, Zhijuan Kang, Bojiao Zuoyuan, Zuocheng Yang

**Affiliations:** Department of Pediatrics, Third Xiangya Hospital of Central South University, 410013 Changsha, Hunan People’s Republic of China

**Keywords:** Diseases, Cardiomyopathies

## Abstract

CVB3 is a single positive-strand enterovirus, and a common pathogen in myocarditis etiology. Although a number of antiviral candidates are under development, specific targeted therapy is not available for CVB3. Ferroptosis is a new type of regulatory cell death discovered in recent years. In this study, our team provided the first evidence that ferroptosis existed in CVB3 infection in vivo and in vitro by iron overload, and massive accumulation of lipid peroxides. Mechanistically, we construct a classical model of HeLa cells following a time-course infection (6, 12, 24, 36, 48 h) with CVB3 (MOI = 10). We demonstrated that the *TFRC* gene plays an important role in promoting ferroptosis in CVB3 infection and downregulation of *TFRC* attenuated the ferroptosis. Interestingly, we observed that *TFRC* was nuclear translocation induced by the CVB3, which was predominantly localized in the cell membrane, but redistributed to the nucleus during CVB3 infection. Moreover, we found that the transcription factor *Sp1* was an essential factor that could bind to the *TFRC* promoter and upregulate the *TFRC* transcription. Collectively, these results suggest that the Sp1/TFRC/Fe axis may provide a new target for the development of therapies against CVB3 infection.

## Introduction

Viral myocarditis is characterized as localized or diffuse disease of the myocardial parenchyma or interstitium caused by virus infection. After coxsackievirus B3 (CVB3) was first used to induce myocarditis in mice in 1974 by Woodruff, most of the subsequent experimental models of viral myocarditis were induced by CVB3 infection [1]. CVB3 is a nonenveloped single-stranded RNA virus belonging to the genus *Enterovirus* of the picornavirus family [2]. It attacks cardiomyocytes by binding to coxsackievirus and adenovirus receptor (CAR) on the membrane, triggering myocarditis by inducing viral replication inside the host cell [3]. Currently, the methods for prevention and treatment of coxsackievirus infection are limited. Hence, people are constantly looking for new mechanisms to inhibit CVB3 damage to host cells. A recent study reported that iron as an essential trace element, played a vital role in inhibition of CVB3 multiplication, thus alterations in trace elements could alter virulence [4]. Iron is a requisite metal in almost all biological systems. However, the level of iron in the cell must be tightly regulated, excess iron accumulate in cells would generate ROS through the Fenton reaction, thereby inducing cell death and global oxidative damage [5].

Ferroptosis is a new type of cell death identified by Stockwell and colleagues, and is characterized by the accumulation of iron (Fe)-dependent reactive oxygen species (ROS) causing lipid peroxidation related cell death [6]. Emerging evidence has identified vital roles of ferroptosis in the pathogenesis of myocardial injury. A clinical study showed that iron overload can trigger heart failure by disturbing iron homeostasis in cardiomyocytes [7]. Another study showed that cardiac injury and hypertrophy caused by ischemia/reperfusion can be significantly mitigated by ferroptosis inhibitors, such as ferrostatin-1 and dexrazoxane, possibly by improving mitochondrial function [8].

*TFRC* encodes the transferrin receptor protein 1 (TFR1, UniProt-P02786) in human, which controls the level of intracellular iron levels. TFR1 imports iron from the extracellular environment into cells, contributing to the cellular iron pool and plays a key role in ferroptosis [9].

Xu et al. [10] found that mice lacking TFR1 in the heart exhibit cardiomegaly, poor cardiac function, failure of mitochondrial respiration, and ineffective mitophagy. Another recent study demonstrated that higher expression of TFR1 in cardiomyocytes was correlated with augmented inflammation in myocarditis patients [11]. However, whether TFR1 is related to CVB3 virus infection has not been reported. As a dynamic process, CVB3 myocarditis involves complex interactions between viruses and host cells, and whether ferroptosis participates in CVB3-induced myocardial injury and the role of *TFRC* in CVB3 infection deserve deep in search.

For the past few years, several cell death including apoptosis, autophagy have been proved to be responsible for CVB3-induced cell death [12, 13]. While the role of ferroptosis in CVB3 infection is still a virgin ground. In this study, we showed that ferroptosis participated in CVB3-induced myocarditis by iron overload, accumulation of lipid peroxides, and mitochondrial morphological change in mice myocardial. Moreover, we demonstrated that *TFRC* is the key gene to regulate the iron overload in CVB3 infection mode, which lead to ferroptosis. Intriguingly, we found that CVB3-induced cell death could be effectively attenuated by Fer-1 and DFO, suggesting that ferroptosis played a important role in CVB3-induced cell death. This study may shed a new light on effective treatments for CVB3-induced cardiac injury.

## Materials and methods

### Animal model and experimental groups

Twelve 4-week-old male Balb/c mice (Department of experimental zoology, Central South University, Changsha, China) weighing 18–22 g were randomly divided into two groups: Control group and CVB3 group (*n* = 6). The mice were blinded in the groups during the experiment. CVB3 group mice were injected with intraperitoneal injection of diluted CVB3 virus solution (dissolved in 0.1 mL normal saline) at 10^3^ TCID50 for one dose, while control group mice received the same volume of normal saline. On day 7 postinfection, all the animals were killed under deep anesthesia. Blood samples were collected for ELISA. Hearts tissue from each group were collected for morphological, biochemical, and molecular analysis. Death during or after injection, and no inflammatory cell infiltration in the myocardium of the infected group mice were excluded.

The animals used in this procedures were following the guide of the national regulations on the administration of experimental animals and have passed the ethical review of experimental animals in Central South University (Acceptance number: No.2021sydw0104).

### Hematoxylin and eosin (HE) staining

Histologic evaluation were used to observe the inflammatory morphological changes in cardiac tissue. In brief, collected heart were fixed in 4% paraformaldehyde, paraffin-embedded and cut into 4 μm thick slices. The sections were deparaffinized with xylene and then rehydrated with ethanol. After staining, sections were sealed with neutral balsam and observed under an optical microscope.

### Transmission electron microscopy (TEM)

TEM was used for observing the morphology of mitochondria and virus particles of heart samples. Samples were fixed in 2.5% glutaraldehyde for 12 h before being washed and post-fixed for 2 h at room temperature in 1% osmium tetroxide. Sections were dehydrated in a graded alcohol series then embedded in pure epoxy resin for 12 h at 40 °C and cured for 48 h at 60 °C. Sections were then sliced, mounted and stained with uranyl acetate and lead citrate, and then viewed on JEM 1400 electron microscope (Japan).

### ELISA assay

Expression level of myocardial injury in myocarditis were evaluated by creatine kinase isoenzyme B (CK-MB) and cardiac troponin I (cTnI) in mice serum using ELISA kit (Jiangsu Jingmei Biological Technology Co., Ltd., China. JM03084M; JM05222H). Blood samples from mice orbital were collected and separated into serum. Taken the 96 well plate coated with antibody from the sealed box. Blank holes and sample holes were tested, respectively. Forty microliters of sample diluent, 10 µL samples, and 100 µL enzyme labeling reagents per well were added in order. The plates were incubated at 37 °C for 60 min and washed carefully for five times before using chromogenic reagent. The reaction was stopped and the wavelength was at OD 450 nm on the microplate reader.

### Cell culture

HeLa cells were obtained from the Institute of Oncology, Central South University, China. The cell line was recently authenticatd and tested for no contamination. Cells were grown in Dulbecco’s modified Eagle’s medium (DMEM, Catalog number 11965, Invitrogen Life Technologies, USA) supplemented with 10% fetal bovine serum (FBS), and incubated at 37 °C in a humidified incubator with 5% CO_2_. The CVB3 Nancy strain was obtained from the Shanghai JiaoTong University School of Medicine and stored at −80 °C in our laboratory. HeLa cells were incubated with CVB3 at an MOI of 10 for 1 h in serum-free DMEM. Then, the cells were washed with phosphate-buffered saline (PBS) and replenished with fresh DMEM containing 10% FBS. HeLa cells without CVB3 infection were used for the mock group. Cells were harvested at different times of CVB3 infection (6, 12, 24, 36, 48 h) as CVB3 groups for subsequent experiments. Each group had three multiples, and each experiment were repeated three independent times.

### Plaque assay

The virus titer in HeLa cells was routinely determined by assessing plaque-forming units (PFU). HeLa cells in six-well plates were infected with a stock solution of 10^5^ PFU/mL, diluted with PBS to produce different working solutions in a gradient. Virus was plaque-assayed in low melting point agarose (overlaid 1.25%) in HeLa cells and stained with crystal violet at 72 h postinfection. Multiplicity of infection (MOI) = (total PFU)/(cell/mL) = 5 × 10^4^ PFU/mL.

### Drug treatment

Erastin (Catalog number S7242, Selleck, TX, USA), a ferroptosis inducer, was diluted with dimethyl sulfoxide (DMSO) to obtain different working solutions and added to the cell medium at different concentrations 12 h before CVB3 infection. Ferrostatin-1 (Fer-1, catalog number S7243, Selleck, TX, USA), an effective lipid ROS scavenger, has been used as a classic ferroptosis inhibitor. Deferoxamine mesylate (DFO, catalog number S5742, Selleck, TX, USA) is a chelator that inhibits ferroptosis. The two inhibitors were diluted in DMSO and used to pretreat cells for 24 h before CVB3 infection at different concentrations.

### Small-interfering RNA and cell transfection

RiboBio genOFF™ siRNAs against human TfR1 and SP1 were purchased from Guangzhou RiboBio Co. Ltd (Catalog number R10043.8, Guangzhou, China). The small-interferring RNA (siRNA) oligonucleotide sequences are shown in Table 1. HeLa cells were transfected with the oligonucleotides at a final concentration of 30 nmol/L. The cells were transiently transfected with the plasmids pcDNA3.1, pcDNA3.1-SP1, pGL3-Basic, and pGL3-Basic-TFRCp and siRNA using Lipofectamine 3000 reagent (Catalog number L3000075, Invitrogen Life Technologies, USA) according to the manufacturer’s protocol. The cells were harvested 48 h after transfection and subjected to follow-up experiments.

**Table 1:** Sequences in genes interference.

Gene number	Target sequence
genOFFTM st-h-TFRC_001	GTAGGATGGTAACCTCAGA
genOFFTM st-h-TFRC_002	GCACAGCTCTCCTATTGAA
genOFFTM st-h-TFRC_003	GGAGACTTCTTCCGTGCTA
genOFFTM st-h-SP1_001	GCAACATCATTGCTGCTAT
genOFFTM st-h-SP1_002	GCCAATAGCTACTCAACTA
genOFFTM st-h-SP1_003	CTCCCAACTTACAGAACC

The sequences of small-interferring RNA (siRNA) oligonucleotide of Human. Each gene (TFRC and SP1) were designed with three interfering target sequences, which was verified by q-PCR and western-blot.

### Cell viability

HeLa cells were seeded in 96‐well plates (5 × 10^3^ cells/well). After 24 h, when the cells grew into a monolayer, they were infected with CVB3 and were harvested at different times. Cell Counting Kit-8 (CCK-8) solution (Catalog number CK04, Dojindo, Japan) was added to each well and incubated with the cells at 37 °C for 1 h. To evaluate cell viability, the optical density at 450 nm was detected using a microplate reader (BIO-RAD Instruments, USA). Cell viability was normalized to the relative control.

### Iron assay

An iron assay kit (Catalog number DIFE-250, BioAssay Systems, USA) was used to quantify the total iron concentration in cell suspensions according to the manufacturer’s instructions. Briefly, tissue (10 mg) or cells (2 × 10^6^/mL) were rapidly homogenized in 4–10 volumes of Iron Assay buffer. The sample was centrifuged at 16,000 × *g* for 10 min at 4 °C to remove insoluble material. Working reagent was added by mixing 20 vol of reagent A, 1 vol of water, and 1 vol of reagent C. Next, 200 μL/well iron probe solution was added and incubated with the samples at room temperature for 40 min, protected from light. Optical density was read at a wavelength of 590 nm.

### Determination of malondialdehyde (MDA)

MDA content was determined with a Lipid Peroxidation MDA Assay Kit (Catalog number S0131, Beyotime, Nanjing, China). Myocardial tissue was homogenized with lysate, centrifuged at 12,000 × *g* for 10 min, and the supernatant was taken. The concentration of both tissue protein and HeLa cell protein were measured using BCA method. The supernatants were blended with thiobarbituric acid detection solution and transferred to 96-well plates to measure the absorbance at 532 nm, and then, the MDA content was calculated according to a standard curve.

### Glutathione assay (GSH)

The intracellular GSH levels were determined using the GSH assay kit (Catalog number S0053, Beyotime, Nanjing, China). The heart tissue were frozen with liquid nitrogen, and then grinded it into powder. To this, 30 μL of removal reagent M solution vortex were added, and then 70 μL of reagent M solution were added and fully homogenized. The sample was centrifuged at 10,000 × *g* 4 °C for 10 min, and the supernatant was used for total glutathione. A total of 2 × 10^5^ HeLa cells were seeded in six‐well plates. Cells were harvested and counted, and GSH levels were measured using GSH assay kit following the manufacturer’s recommendations. The absorbance value was measured at 412 nm on the microplate reader.

### Lipid peroxidation assay

BODIPY™ 581/591 (Catalog number D3861, C11-BODIPY, Invitrogen, USA) probes that change their fluorescence properties upon oxidation. It shifts fluorescence of the dye from red signals (581/610 nm) to green signals (484/510 nm). Oxidation of the polyunsaturated butadienyl portion of C11-BODIPY results in a shift of the fluorescence emission peak from ~590 to ~510 nm. C11- BODIPY resides in lipophilic membrane structures where it can be oxidized by reactive enzymatic and/or non-enzymatic pro-oxidant intermediates, including alkoxyl and peroxyl radicals. Its oxidation is therefore an indirect manifestation of the lipid ROS flux, further coined as lipid ROS [11]. The methods used are according to the manufacturer’s instructions. Briefly, cells were seeded on glass coverslips, infected with CVB3 at an MOI of 10 for 3 h and then incubated with the C11- BODIPY™ 581/591 probe for 30 min. After three washes with 1 × PBS, cells were stained with a 0.1 μg/mL solution of DAPI dye. Fluorescence signals were visualized with an inverted fluorescence microscope system (OLYMPUS-IX71 systems, Japan). Analysis of images was performed using ImageJ software. Positive signal of immunofluoresce were calculated by ImageJ software, the details are as follows: 1. Split a single channel after opening C11-BODIPY fluorescence images; 2. Change the type of images color from RGB to RGB stack; 3. Control the contrast and adjust threshold; 4. Change the B&W on the right of default to red; 5. Analyze and set measurement. Area fraction (%Area) is the area occupied by positive fluorescent signal.

### Immunofluorescence assay (IFA)

HeLa cells were seeded in 20-mm glass-bottomed dishes. After infection for 6, 12, 24, 36, and 48 h, the cells were fixed with 4% paraformaldehyde in PBS for 30 min and permeabilized with 0.5% Triton X-100 in PBS for 10 min. The cells were then incubated at 4 °C overnight with mouse anti-enterovirus mAb (Catalog number LS-C744297, 1:50, Lifespan) or anti-TFRC mAb (Catalog number ab214039, 1:100, Abcam, UK) as the primary antibodies. After three washes with PBS, the cells were incubated with secondary goat anti-rabbit IgG Alexa Fluor 488 (Catalog number ab150077, 1:200, Abcam) at 37 °C for 1 h. Cell nuclei were stained with DAPI for 10 min at room temperature, and fluorescence signals were visualized with an inverted fluorescence microscope system (OLYMPUS-IX71 systems, Japan). Analysis of images was performed using ImageJ software.

### Real‐time polymerase chain reaction

Total RNA was isolated and purified using TRIzol reagent (Catalog number 15596018, Invitrogen) according to the manufacturer’s protocol. RNA was reverse transcribed using a Hiscript RT SuperMix kit (Catalog number R222-01, Vazyme, Nanjing, China). mRNA expression was assessed using an Applied Biosystems Prism 7300 sequence detection system with ChamQ Universal SYBR qPCR Master Mix (Catalog number Q711-02/03, Vazyme, Nanjing, China) according to the manual, with β-actin as the internal normalized reference. The relative quantification was calculated using the 2^−ΔΔCt^ cycle threshold method. Primer sequences were obtained from the human GRCh38 UCSC Genome Database and designed with Primer 3 software (Tables 2 and 3), and then, BLAST was used for sequence alignment.

**Table 2:** Primers in experiments in HeLa cells.

Primer name	Primer sequence(5’-3’)	Tm	Product size
β-actin	F:CATGGAGTCCTGTGGCATC	59 °C	157 bp
	R:CAGGGCAGTGATCTCCTTCT		
ACSL4	F:AATGCAGCCAAATGGAAAAG	60 °C	152 bp
	R:CACAGAAGATGGCAATGGTG		
GPX4	F:CAGTGAGGCAAGACCGAAGT	59 °C	111 bp
	R:CTGCTTCCCGAACTGGTTAC		
NCOA4	F:GCACTTGATGGCTCATGCTA	60 °C	151 bp
	R:ATAACCACTGGCAGGTTTGC		
Fth1	F:TGACAAAAATGACCCCCATT	59 °C	160 bp
	R:CAGGGTGTGCTTGTCAAAGA		
TFRC	F:AAAATCCGGTGTAGGCACAG	59 °C	179 bp
	R:TTAAATGCAGGGACGAAAGG		
SP1	F:TCATACCAGGTGCAAACCAA	60 °C	224 bp
	R:GCTGGGAGTCAAGGTAGCTG		
MAVS	F:CCTAAGGCCCTCTCTTTGCT	59 °C	185 bp
	R:GCACCTCCAAAGAGCTTGAC		
TFAP2A	F:ACTGAGACTCCCGTCAATGG	60 °C	231 bp
	R:GCGTGTTCCTTAATCCGTGT		
STAT3	F:CTGGCCTTTGGTGTTGAAAT	59 °C	202 bp
	R:AAGGCACCCACAGAAACAAC		
TFRCp-BS1	F:GAGCCCAGGAGTTCAAGACTA	58 °C	150 bp
	R:ATTCCTGACCTCAGGTGATCT		
TFRCp-BS2	F:TACGTGCCTCAGGAAGTGAC	5 °C	184 bp
	R:AGTGGCAGAAACAGTGGATG		
TFRCp-BS3	F:GTACGTGCCTCAGGAAGTGA	58°C	150 bp
	R:GAAATGACAACGAGGGGATG		

The primer of gene’s sequence, annealing temperature, and product size in PCR in HeLa cells.

**Table 3:** Primers in experiments in Mice.

Primer name	Primer sequence(5’–3’)	Tm	Product size
β-actin	F: GCTACAGCTTCACCACCACA	58 °C	208 bp
	R:AAGGAAGGCTGGAAAAGAGC		
CVB3	F:GGCGCTAGCACTCTGGTATC	59 °C	191 bp
	R:CGAACGCTTTCTCCTTCAAC		

The primer of gene’s sequence, annealing temperature, and product size in PCR in mice heart tissue.

### Western blot analysis

Protein samples were extracted from HeLa cells with RIPA lysis buffer (Catalog number P0013B, Beyotime, Shanghai, China) and quantified using Pierce BCA Protein Assay Kit (Catalog number 23225, Thermo Fisher Scientific). Target proteins were transferred to immobilon-P PVDF membranes (IPVH00010, Millipore, Merck, Germany) after separation via 10% sodium dodecyl sulfate polyacrylamide gel electrophoresis. Protein bands were incubated with primary antibodies and paired secondary antibodies. The Tanon‐5200 Imaging System (Tanon, Shanghai, China) was used to analyze protein levels. Antibodies targeting the following proteins were used ACSL4 (Catalog number ab155282, 1:1000, Abcam), GPX4 (Catalog number 67763-1-Ig, 1:1000, Proteintech), Fth1(Catalog number A1144, 1:500, ABclonal), NCOA4 (Catalog number A5695, 1:500, ABclonal), SLC7A11/xCT (Catalog number ab175186, 1:1000, Abcam), TFR1 (Catalog number ab214039, 1:1000, Abcam), SP1 (Catalog number ab231778, ChIP Grade, diluted 1:1000, Abcam), and β-actin (Catalog number 20536-1-AP, 1:1000, Proteintech) as internal parameter.

### Subcellular fractionation analysis

HeLa cells were seeded in 10-cm glass-bottomed dishes. After CVB3 infection for 12, 24, 36, and 48 h, the cells were harvested. Nuclear protein was extracted using Nucleoprotein Extraction Kit (Catalog number C500009, Sangon Biotec, Shanghai, China). According to the instructions, removing the culture medium of adherent cultured cells, 10 mL of cooled 1x PBS rinsing solution were added to the 100 mm culture dish for washing twice, and the cells were scraped off . Next, the cells were transferred to centrifuge tubes and centrifuged at 800 × *g* at 4 °C for 10 min, the prepared cooled hypotonic buffer were added. The sediment was suspended, ice bath for 10 min, shake for 10 s, and mix evenly. Finally, the suspension was centrifuged and the supernatant was the cytoplasm protein, which were transferred to clean cooled tubes. Then, 0.2 mL of lysis buffer were added to the precipitation, centrifuged at 3000 × *g* at 4 °C for 10 min, the supernatant was the nuclear protein extract. The membrane protein was extracted using Membrane and Cytosol Protein Extraction Kit (Catalog number P0033, Beyotime, China). The HeLa cells were washed with cooled PBS and the cells were scraped off. Then it was centrifuged and resuspended with PBS, 1 mL of membrane protein extraction reagent was added with PMSF to 20–50 million cells, gently and fully suspend the cells, it was then placed in ice bath for 10–15 min. Centrifuge suspension at 4 °C, 700 × *g* for 10 min, collected the supernatant into a new centrifuge tube to remove nuclei and unbroken cells. Next, centrifugation 4 °C, 14,000 × *g* for 30 min, the absorbed supernatant is the cytoplasmic protein. Adding 0.2 mL of extraction reagent B, vortexed for 5 s, re-suspension precipitation, ice bath for 5–10 min. Then, centrifuge at 4 °C, 14,000 × *g* for 5 min, and collect the supernatant as the cell membrane protein solution. Using Pierce BCA Protein Assay Kit to quantify the protein, and Western-blot was used to measure the expression of TFR1 in nuclear, membrane and cytoplasm. The internal parameters for cell fractionations: Lamin b1 (proteintech, 66095-1-Ig) for nuclear protein, Na, K-ATPase (CST, 3010) for membrane protein, β-actin (proteintech, 66009-1-Ig) for cytoplasm protein.

### Luciferase reporter assay

The *TFRC* promoter sequence was cloned into a pGL3-basic vector (Catalog number E1751, Promega, USA) and used to transfect target cells using Lipofectamine 3000. At 48 h posttransfection, cells were harvested and the cell lysate was analyzed using a Dual-Luciferase Reporter Assay System (Catalog number E1910, Promega, USA). The ratio of firefly luciferase activity to Renilla luciferase activity in each sample served as a measure of normalized luciferase activity.

### Chromatin immunoprecipitation (ChIP) assay

ChIP assays were carried out using a Merck-ChIP Kit (Catalog number #17–371, Millipore, Merck, Germany) according to the manufacturer’s protocol. Briefly, cells were grown to 90% confluence in three 10 cm culture dishes and crosslinked with 1% formaldehyde at room temperature for 10 min. Glycine was used to quench excess formaldehyde, and the cells were scraped and pelleted. Chromatin was sonicated in lysis buffer on ice to 200–1000 bp using a sonicator (Sonics, USA) at 55–75% intensity four times. The sheared DNA lengths were confirmed via agarose gel electrophoresis. After sonication, the chromatin was incubated with an anti-SP1 antibody (Catalog number ab231778, ChIP Grade, Abcam) or IgG and rotated at 4 °C overnight. Reverse crosslinking of the protein–DNA complexes was performed according to the manufacturer’s protocol. Spin columns were used for ChIP DNA extraction. Standard PCR was performed, and products were detected via 3% agarose gel electrophoresis and visualized using a Syngene Bio Imaging system. The PCR primers used to amplify the promoter region are provided in Table 2. Potential binding sites (BS) for Sp1 within the promoter region of TFRC were identified using JASPAR (http://jaspar.genereg.net/).

### Statistical analysis

Assays for characterizing cell phenotypes were analyzed using Student’s *t*-test, and correlations between groups were calculated using Pearson’s test. ANOVA was used for comparison among groups. Observe between the inter group variance and intra group variance. The ratio of iner group variance was relatively small. Differences with *p* < 0.05 were deemed statistically significant. Data were analyzed using GraphPad Prism 8 software (GraphPad Software, La Jolla, CA, USA).

## Results

### Ferroptosis is exist in acute CVB3-induced viral myocarditis

**Fig. 1:**
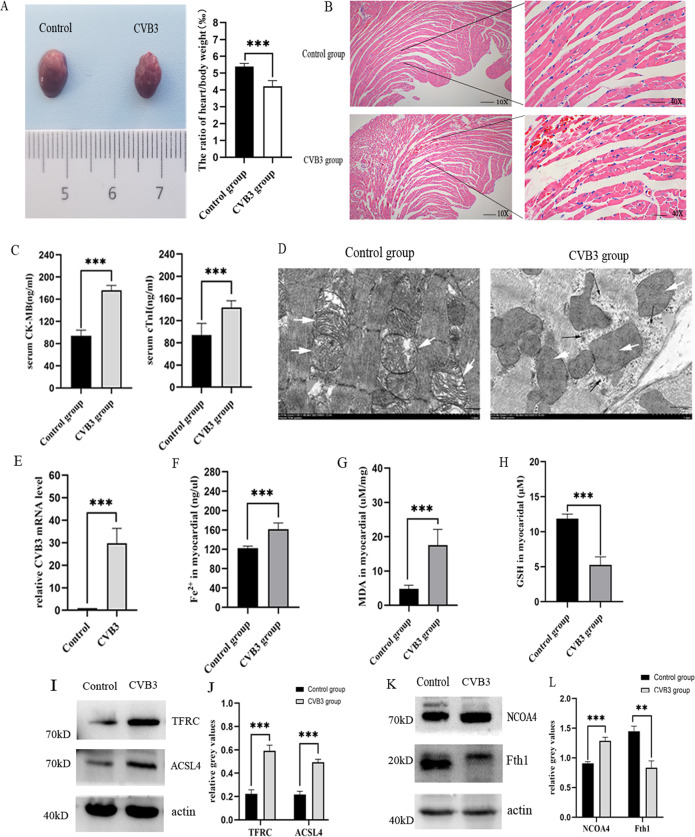
Ferroptosis exist in CVB3-induced myocardial injury in vivo. **A** General basic morphology of the heart in the normal control group and the CVB3-infected group in Balb/c mice. The ratio of heat weight compared to body weight in two groups was shown in the statistical chart. *n* = 6. **B** The representative pictures of H&E staining in control group and CVB3 infection group mice myocardial (scale bar = 50 μm). **C** The chart represented the comparison of serum myocarditis inflammation indexes CK-MB and cTnI in control and CVB3 group mice. *n* = 6. **D** Transmission electron microscopy were used to detect the mitochondrial morphology and virus particles of myocardial tissue in control and CVB3 mice. The white arrow indicated mitochondria, and the black arrow indicated virus particles in CVB3 group (scale bar = 1 μm). **E** The level of CVB3 virus mRNA in myocardial was detected by qPCR. *n* = 6. **F** Iron concentrations were measured in the control and CVB3 groups using an Iron Assay Kit. *n* = 6. **G** MDA concentrations in the two groups myocardial were determined by Lipid Peroxidation MDA Assay Kit. *n* = 6. **H** GSH in myocardial tissue were detected using the GSH assay kit. *n* = 6. **I**, **J** Representative images of western-blotting and relative gray values on ferroptosis genes TFRC and ACSL4 protein expression. **K**, **L** Images of NCOA4 and Fth1 protein expression and relative gray values by western-blotting and Image J. *n* = 6. All results are expressed as the mean ± SD. *t*-test was used for comparison between two groups. *p < 0.05, **p < 0.01, ***p < 0.001.

To explore whether ferroptosis participate in CVB3-induced myocardial injury, we first generated the acute viral myocarditis model by intraperitoneal injection of 10^3^ TCID50 of CVB3 into 4-week-old male Balb/c mice as reported in literature [14]. Mice were sacrificed on day 7 postinfection, the photo of general morphological change in CVB3 group showed that the volume of heart was reduced, the myocardial surface became uneven, and the ratio of heart to body weight of mice in the infection group decreased significantly compared to control group (*p* < 0.001) (Fig. 1A). Hematoxylin and eosin (HE) staining showed apparent inflammatory infiltrate in myocardial fibers and perivascular tissue (Fig. 1B), and the sensitive indicators of myocardial injury serum creatine kinase myocardial band (CK-MB) and cardiac troponin I (cTnI) were higher in CVB3 group than that in control group (*p* < 0.001) (Fig. 1C). To confirm that the pathogen of myocarditis, we found the viral particles in myocardial tissue by transmission electron microscopy (TEM) (Fig. 1D), and the CVB3 mRNA level was increased sharply in CVB3-infected myocardial using q-PCR (Fig. 1E). All these results suggest that the successful establishment of CVB3-induced myocarditis.

Next, we detected the symbol representatives of ferroptosis. Ferroptosis is characterized by the morphological change in mitochondrial, accumulation of iron, and an abnormal surplus of peroxide metabolites [15, 16]. TEM in our study showed that disrupted cristae and condensed membrane densities in mitochondrial in CVB3 group (Fig. 1D). As shown in Fig. 1F, the level of ferrous iron was significantly increased in CVB3-infected mice myocardial. Besides, MDA as a second metabolite product of lipid peroxide was also significantly enhanced in CVB3 group (*p* < 0.001) (Fig. 1G). GSH as an important antioxidant was decreased after CVB3 infection (Fig. 1H), the depletion of which was confirmed in ferroptosis [17, 18]. To explore the possible causes of iron elevation in tissue, we detected the main genes related to iron metabolism in vivo. We found that in CVB3 group, the increase of upregulated iron genes TFRC and NCOA4 were elevated (Fig. 1I, K), especially TFRC was almost three times than control group, and downregulated iron gene Fth1 was decreased in CVB3-infected myocardial (Fig. 1K). In additon, the expression of ACSL4 that contributes to ultimate ferroptosis was also increased significantly in CVB3 group (Fig. 1I). Taken together, these data demonstrate that ferroptosis is present in acute CVB3-induced myocarditis.

### Ferroptosis is activated in CVB3-infected HeLa cell

**Fig. 2:**
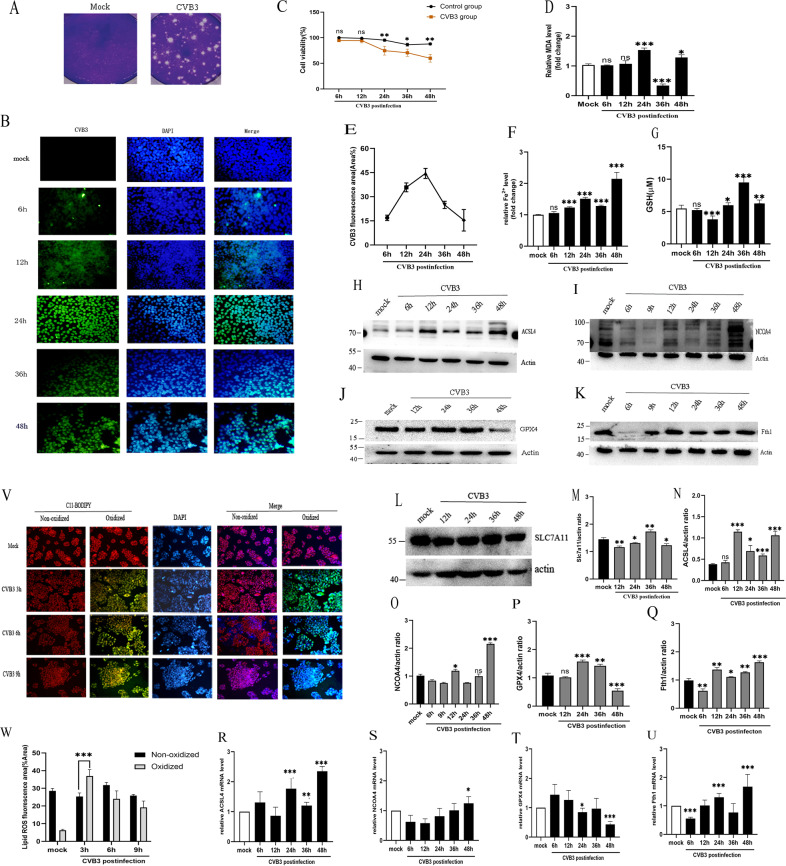
Ferroptosis participates in CVB3-induced cell death in vitro. **A** Plaque morphology of the CVB3 virus in HeLa cells. Viral titers in HeLa cells were determined as plaque-forming units (PFU) (right). The mock group contained non-infected HeLa cells (left). **B** Detection of anti-enterovirus antibody (green) via IFA at 6, 12, 24, 36, 48 h post-CVB3 infection in HeLa cells (scale bar = 50 μm). Cell nuclei were stained with DAPI (blue). **C** The cell viability in CVB3-infected groups was measured with CCK-8 assays at OD 450 nm. **D** MDA concentrations were determined with Lipid Peroxidation MDA Assay Kit at a wavelength of 532 nm. MDA in the CVB3 group was increased significantly compared to the mock group at 24 h infection. **E** Area of CVB3-positive fluorescence were measured with ImageJ. **F** Iron concentrations were determined with an Iron Assay Kit at a wavelength of 593 nm. Iron in the CVB3 group was two times greater than that in the control group at 48 h post-CVB3 infection. **G** Reduced glutathione (GSH) in HeLa cells were detected using the GSH assay kit and measured the absorbance value at 412 nm on the microplate reader. **H**–**L** The expression of protein levels in ferroptosis genes were measured via western-blotting. **M**–**Q** The corresponding statistical charts of relative gray values in accord with western-blot strips. **R**–**U** The mRNA levels of ferroptosis genes were detected via qPCR. **V** Representative fluorescence images of BODIPY581/591C11 (2 μM, incubate for 30 min) staining of HeLa cells infected with CVB3 for 3, 6, and 9 h (scale bar = 100 μm). BODIPY™ 581/591 probes change their fluorescence properties upon lipid oxidation. Oxidized cells with Lipid ROS were emission green signals (484/510 nm), while none oxidized cells were appeared red signals (581/610 nm). **W** Percentage of oxidized/non-oxidized cells were calculated by ImageJ software according to figure **V**. It indicated that lipid ROS appeared after CVB3 infection at 3 h of early stage. All results are expressed as the mean ± SD. *p < 0.05 vs. the mock group, **0.01 < *p* < 0.05, ***p < 0.01. *n* = 3.

HeLa cells are highly susceptible to virus and are widely used for classical model to study the cell death of CVB3 infection like apoptosis and autophagy [19]. To explore the mechanism of ferroptosis induced by CVB3, we first did a plaque assay to calculate the viral titer, it was 10 MOI (5 × 10^5^ PFU/mL) in HeLa cell with CVB3 infection (Fig. 2A). Then, we used IFA to observe virus replication in HeLa cells at different stages of infection (Fig. 2B, E). The results showed that virus replication in cells began at 12 h, and the largest virus amount was observed 24 h after infection (Fig. 2B), which was accord with the report that human-induced pluripotent stem cells (hiPSCs) also displayed an increase in CVB3-Luc infection at 24 h infection [20]. These results indicated that the successful construction of the CVB3 infection model in vitro.

Next, we performed CCK-8 assay to evaluate of virus on cell viability, it was found that the viability of the CVB3-infected cells was decreased 24 h after CVB3 infection, by 48 h, the cell death number was close to 50% (Fig. 2C). To determine the activation of ferroptosis, we set and tested ferroptosis markers in different periods. At first, we measured the normal control groups (Mock group) at each time point. The results showed that intracellular iron, MDA, and GSH had no significant difference between the mock groups (Supplementary Fig. 1A–C) (*p* > 0.05). Besides, the mRNA (Supplementary Fig. 1D–H) and protein (Supplementary Fig. 1I, J) of recognized pro-ferroptosis genes TFRC, ACSL4 and anti-ferroptosis SLC7A11, GPX4, Fth1 also had no obvious variations between groups (*p* > 0.05). However, in CVB3-infected groups, the intracellular iron increased from 12 h after CVB3 infection and reached the maximum value at 48 h (Fig. 2F). MDA increased at 24 h postinfection (Fig. 2D). Interestingly, GSH level was first decreased at early CVB3 infection, however, it increased from 24 h postinfection and reached the maximum value at 36 h (Fig. 2G). BODIPY-C11 detects the early reactive oxygen species (ROS) in a lipophilic environment through a change in the fluorescence of the probe [21]. It showed that an overwhelming level of lipid ROS production was at early infection (3 h postinfection) in the CVB3 group (Fig. 2V, W). Taken together, these results suggested that ferroptosis was activated by CVB3 infection.

In order to determine when ferroptosis occur during CVB3 infection in vitro, we detected the upstream of ferroptosis pathway System X_C_^−^. SLC7A11 is a main composition of System X_C_^−^ [22]. In our study, we found that SLC7A11 expression was decreased at early CVB3 infection but increased obviously at 36 h postinfection (Fig. 2L, M), which was consistent with the level of GSH. However, at 48 h postinfection, the SLC7A11 expresssion declined, another important GSH synthesis enzyme GPX4 was also downregulated (Fig. 2J). To further explore the reason, we measured the change of core regulatory genes in ferroptosis. As shown in Fig. 1H, I, J, the levels of pro-ferroptotic genes ACSL4 and NCOA4 were significantly upregulated at 48 h postinfection, while the negative regulator GPX4 level was downregulated at 48 h (Fig. 2N, O, P). Similar to WB results, the mRNA of ACSL4 and NCOA4 were increased at 48 h postinfection (Fig. 2R, S). Meanwhile, GPX4 mRNA level was reduced (Fig. 2T). Hence, our results has suggested that ferroptosis is occurred mainly at 48 h CVB3 infection in vitro.

### The effect of inhibitors on ferroptosis in CVB3 infection

**Fig. 3:**
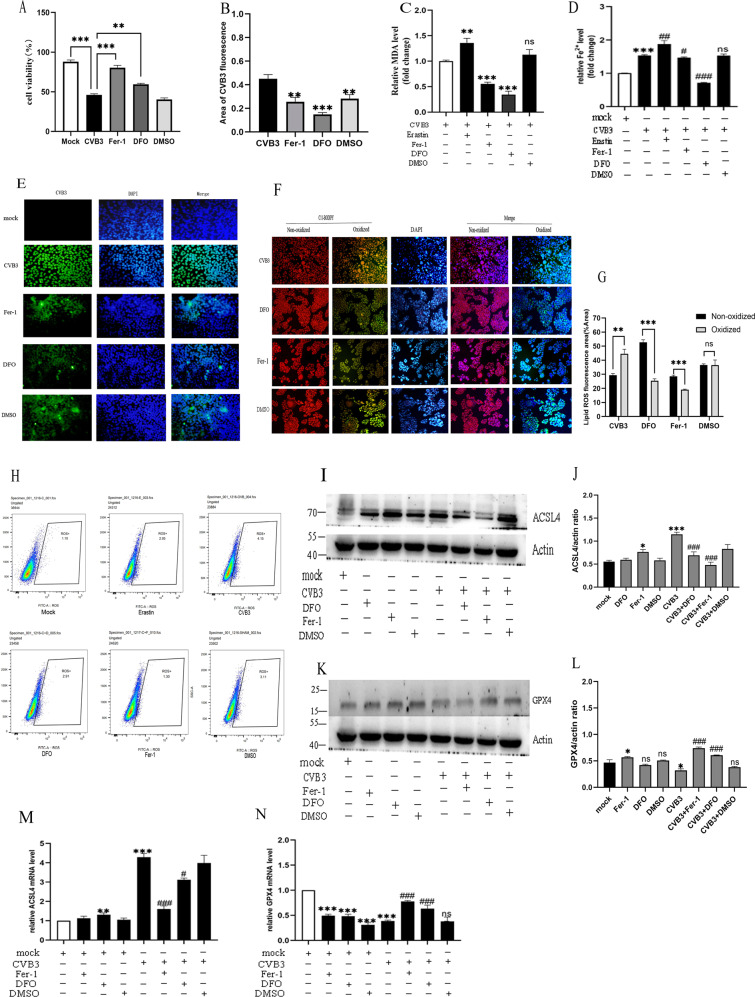
The effect of drugs on ferroptosis in CVB3-infected cells. **A** Cell viability of HeLa cells in different drug treatment groups measured by CCK-8 assays. Fer-1 pretreated at 2 μM, 24 h before CVB3 infection. DFO pretreated at 5 μM 24 h before CVB3 infection. DMSO was used as a solvent control. The results showed that both Fer-1 and DFO could prevent the decrease in cell viability caused by CVB3 infection. **B** Area of CVB3-positive fluorescence measured with ImageJ, which corresponds to figure **E**. **C** MDA detection under different ferroptosis drug interventions. It showed that both Fer-1 and DFO drugs can reduce MDA production, especially DFO (*p* < 0.01). **D** Iron concentrations were measured after pretreatment of CVB3-infected HeLa cells with the ferroptosis agonist Erastin (6 μM, 12 h) and inhibitors Fer-1 and DFO. **E** CVB3 fluorescence (green) determined by IFA in HeLa cells in different drug intervention groups. Cell nuclei were stained with DAPI (blue) (scale bar = 50 μm). **F** Fluorescence of lipid ROS in HeLa cells pretreated with Fer-1 or DFO and incubated with the BODIPY581/591C11 probe (scale bar = 100 μm). **G** Percentage of oxidized/non-oxidized cells were calculated by ImageJ software according to figure **F**. It suggested that both DFO and Fer-1 could decrease the oxidized lipid ROS significantly (*p* < 0.001). **H** Flow cytometry was used to detect lipid ROS in different drug pretreatment groups during CVB3 infection. **I**, **J** The protein level of ACSL4 in drug groups were measured by western-blot, the gray values of strips were calculated by ImageJ. **K**, **L** The protein level of GPX4 in different drug groups were measured by western-blot. Measurement of relative gray values indicating gene protein levels were determined using ImageJ. **M**, **N** The mRNA level of ACSL4 and GPX4 in the drug-pretreated groups were detected via qPCR. All results are expressed as the mean ± SD. *p < 0.05 vs. the mock group, **p < 0.01, ***p < 0.001. #*p* < 0.05 vs. the CVB3 group, ##*p* < 0.01, ###*p* < 0.001. *n* = 3.

To further explore the role of iron in ferroptosis induced by CVB3 infection, we used two classical ferroptosis inhibitors, ferrostatin-1 (Fer-1) and deferoxamine (DFO). We first used the inducer erastin to induce ferroptosis at 6 μM (Supplementary Fig. 2A), then rescue experiments were performed by treating cells with ferroptosis inhibitors Fer-1 (at 2 μM, Supplementary Fig. 2B) or DFO (at 5 μM, Supplementary Fig. 3C) after induction by erastin. As shown in Fig. 3A, both Fer-1 and DFO could rescue the cell viability decrease induced by CVB3 infection, compared to the non-drug intervention CVB3 group (Fer-1 *p* < 0.001, DFO *p* < 0.01).

To compare the effects of the two inhibitors on ferroptosis induced by CVB3 infection, we measured iron content, the MDA level, and lipid ROS levels. The results showed that both Fer-1 and DFO can relive the CVB3 virus replication at 24 h of infection (DFO *p* < 0.001, Fer-1 *p* < 0.01) (Fig. 3B, E). The iron chelator DFO had the most obvious effect on decreasing MDA and iron content (DFO *p* < 0.001, Fer-1 *p* < 0.05). (Fig. 3C, D). Fer-1 had a more obvious effect than DFO on reducing the accumulation of lipid ROS by IFA (Fig. 3F, G) and by flow cytometry (Fig. 3H). Moreover, We detected the changes in the representative ferroptosis genes ACSL4 and GPX4 via qPCR and western-blotting analyses. Both Fer-1 and DFO rescued the mRNA and protein expression of ACSL4 in the CVB3-infected groups (Fig. 3I, J, M), but had no evident effect in non-infected groups, and Fer-1, DFO could also prevent the reducing of GPX4 mRNA and protein levels by CVB3 infection (Fig. 3K, L, N). Taken together, these results indicate that the ferroptosis inhibitors Fer-1 and DFO can both inhibit ferroptosis induced by CVB3 infection.

### Downregulation of *TFRC* inhibited ferroptosis in CVB3-infected HeLa cells

**Fig. 4:**
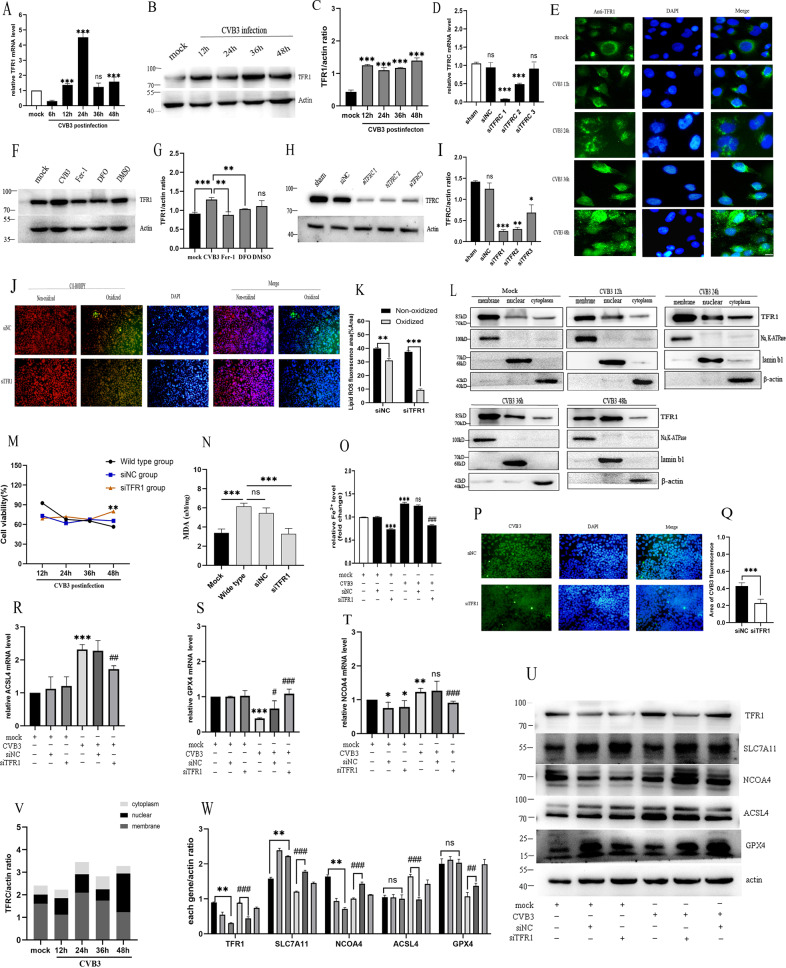
TFR1 is the key gene leading to ferroptosis in CVB3-infected HeLa cells. **A** The mRNA level of *TFR1* at different CVB3 infection times was detected via qPCR. The *TFR1* mRNA increased obviously at 24 h of CVB3 infection (*p* < 0.001). **B**, **C** The protein level of *TFR1* and relative gray values at different CVB3 infection times were measured via western-blotting. **D** *TFR1* knockdown effects on three different sequences at the mRNA level via qPCR. **E** Determination of nuclear localization of *TFR1* by immunofluorescence with CVB3 infection. The results showed that the *TFR1* was translocated from the cell membrane to the nucleus with CVB3 infection. Scale bar = 10 μm. **F**, **G** The effect of ferroptosis drugs on *TFR1* protein expression at 48 h post-CVB3 infection determined by western-blotting. **H**, **I** The image and gray values of *TFR1* knockdown effects on three different sequences at the protein level via western-blotting. **J** Lipid ROS levels in siNC and siTFR1 groups were detected via immunofluorescence with BODIPY581/591C11 (scale bar = 100 μm). **K** The statistical results showed that the lipid ROS decreased significantly in siTFR1 group compared to siNC group (*p* < 0.001). **L** Subcellular fractionation analysis of *TFR1* expressions in cell membrane, nuclear, cytoplasm using different cell component extraction kits and measured by western-blot. Na,K-ATPase, lamin b1 and β-actin were as internal references for cell membrane, nucleus, and cytoplasm in western-blot, respectively. **M** Cell viability in the siTFR1 group was obviously improved at 48 h post-CVB3 infection, measured by CCK-8 assays. **N** MDA levels were measured by MDA Assay Kit at a wavelength of 532 nm. It showed knockdown of *TFR1* could reduce MDA levels compared to the wide-type group (*p* < 0.001). **O** The iron level in siTFR1 and NC groups were measured using an iron assay. It indicated that *TFR1* knockdown could decrease cellular iron levels obviously. **P**, **Q** Effect of *TFR1* knockdown on CVB3 fluorescence detected by IF. Cell nuclei were stained with DAPI (blue) (scale bar = 50 μm). **R**–**T** The mRNA levels of ferroptosis genes ACSL4, GPX4 and NCOA4 of *TFR1* knockdown via qPCR. **U**, **W** The protein levels of major genes in the siTFR1 groups were detected via western-blotting. Corresponding of relative gray values of proteins were measured using ImageJ. **V** Relative gray values of subcellular fractionation analysis of western-blot strips in figure **L** measured by ImageJ. It suggested that membrane protein TFR1 was mainly located in membrane in mock group, however, it was partially transfered to nuclear induced by CVB3 infection, especially at 48 h postinfection.

*TFRC* gene encoded the protein of TFR1, which is a type 2 membrane protein expressed as a homodimer in the cell membrane that constitute the main component of transferrin (Tf) [23]. In light of the TFR1 level has been demonstrated significantly increased in acute myocarditis patients and associated with augmented inflammation (CRP, IL-6) [11], we hypothesized that *TFRC* might be a key effector gene of pro-ferroptosis in CVB3 infection. To confirm the hypothesis, we searched in KEGG database and screened some candidate genes involved in iron overload-induced ferroptosis, then we analyzed these genes mRNA expression by qPCR (Supplementary Fig. 3A). The analysis identified the *TFRC* gene, which was upregulated more than fourfold in the CVB3-infected group compared to the mock group at 24 h postinfection (Fig. 4A). Consistently, western-blot analysis showed that *TFRC* protein levels were also increased after CVB3 infection (Fig. 4B, C). To evaluate the role of *TFRC* in iron overload, we treated HeLa cells with Fer-1 and DFO. As expected, these two drugs decreased *TFRC* expression detected by western-blot analysis (Fig. 4F, G). Of note, via immunofluorescence, we found that the subcellular localization of *TFRC* after CVB3 infection at different time points (Fig. 4E). *TFRC* began to be observed in the cell membrane. After 24 h of CVB3 infection, it gradually migrated around the nucleus, which was most obvious at 48 h postinfection. To verify this finding, we also did subcellular fractionation analysis using different internal references for nuclear membrane and cytoplasm in western-blot, the results were demonstrated nuclear translocation of *TFRC* in response to CVB infection in Fig. 4L, V. Thus, it could be inferred that *TFRC* may play an important role in CVB3-induced infection.

To confirm the role of *TFRC* in CVB3 infection, we generated *TFRC*-knockdown (*TFRC*-KD) HeLa cells using siRNA. Of the three pairs of siRNAs tested, siTFR1 was the most efficient in knock down of *TFRC* expression, as verified by qPCR and western-blotting analyses (Fig. 4D, H, I). Phenotypic detection of ferroptosis showed that, the siTFR1 group significantly decreased the lipid ROS compared to NC group (Fig. 4J, K). Additionally, the cell viability was improved by siTFR1 when CVB3 infected 48h, which detected by CCK-8 assay (Fig. 4M). MDA was reversed by TFR1 knockdown after CVB3 infection (Fig. 4N). Cellular iron levels was also lower in the siTFR1 group than siNC group (Fig. 4O). Moreover, we assessed the role of *TFRC* knockdown on CVB3 virus replication via IFA, the result revealed that the number of virus-infected cells in the siTFR1 group was significantly lower than that in the NC group at 24 h CVB3 postinfection (*p* < 0.001, Fig. 4P, Q). Next, we examined whether downregulation of *TFRC* affected typical ferroptosis genes through q-PCR and western-blot. As shown in Fig. 4R, U, W, siTFR1 decreased ACSL4 both at the mRNA and protein level, enhanced GPX4 mRNA and protein levels in CVB3-infected group compared to wild-type group (Fig. 4S, U, W). In non-infected HeLa cells, siTFR1 lowered NCOA4 expression but not ACSL4 and GPX4 mRNA expression compared to mock group (Fig. 4T, U, W). SLC7A11 is a negative regulator of ferroptosis and was upregulated at the protein level in the siTFR1 group (Fig. 4U, W). Taken these together, our results has demonstrated that *TFRC* is a key gene in ferroptosis and inhibition of *TFRC* can downregulate ferroptosis in CVB3-infected HeLa cells. Moreover, we found *TFRC* expression was translocated to nuclear with CVB3 infection by IFA and subcellular analysis.

### Sp1 regulates *TFRC* transcription in HeLa cells by binding to the *TFRC* promoter region

**Fig. 5:**
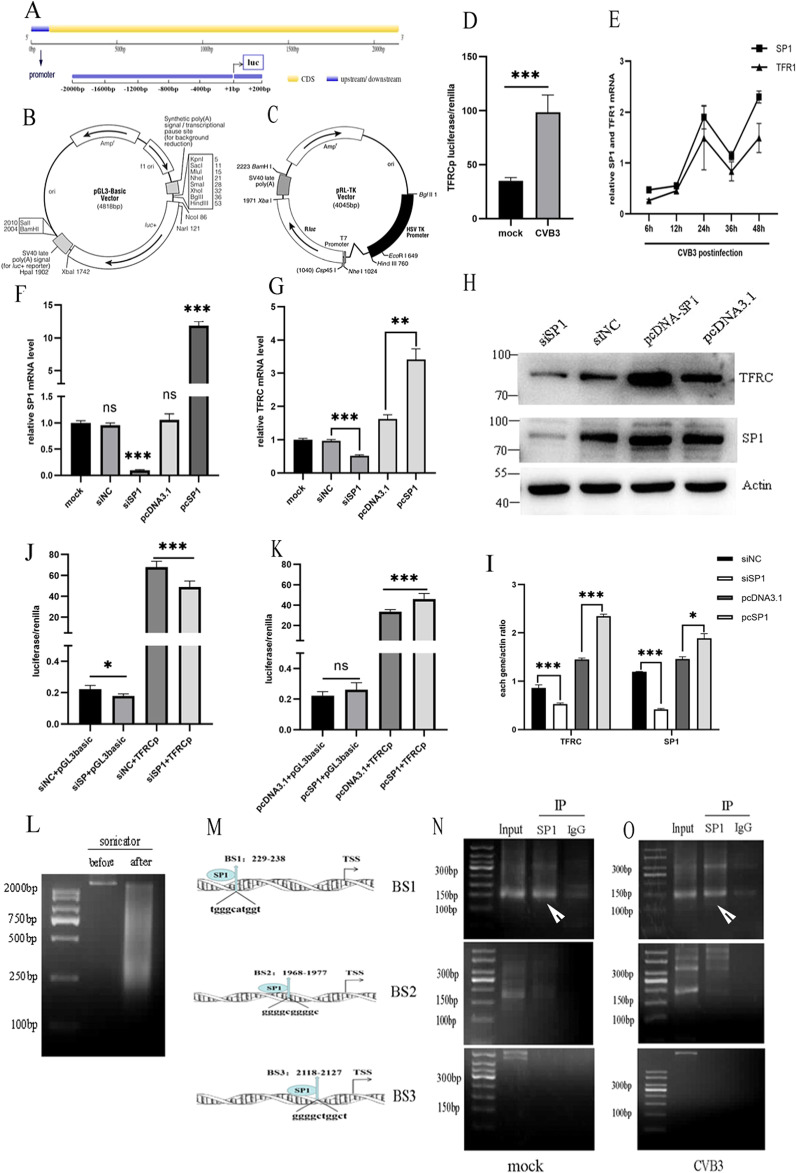
*TFRC* transcriptional regulation. **A** Map of the promoter location in the *TFRC* gene. **B** Construction map of the *TFRC* promoter plasmid. **C** Construction map of the firefly luciferase reporter plasmid. **D** The activity of the *TFRC* promoter in the CVB3 group was significantly higher than that in the mock group based on luciferase reporter assay results. **E** mRNA level of Sp1 was consistent with that of *TFRC* at various CVB3 infection times by qPCR. **F** Effect of Sp1 knockdown and overexpression at the mRNA level determined by qPCR. **G** The influence of Sp1 upregulation and downregulation on *TFRC* mRNA expression via qPCR. **H**, **I** The effect of Sp1 upregulation and downregulation on *TFRC* protein expression. **J** Downregulation of Sp1 reduced the activity of the TFRC promoter by Luciferase Reporter Assay. **K** Upregulation of Sp1 enhanced the activity of TFRC promoter by Luciferase Reporter Assay. **L** Chromatin from HeLa cell lysis buffer was subjected to agarose electrophoresis before and after sonication in the ChIP experiment. **M** The three potential binding sites (BS) for Sp1 within the promoter region of *TFRC* predicted by JASPAR (http://jaspar.genereg.net/). **N**, **O** The binding sites for the transcription factor Sp1 in the *TFRC* promoter were verified via ChIP-PCR agarose gel electrophoresis (white arrows—indicate binding sites). The input group included total chromatin samples, and IgG served as the negative control. In the IP- Sp1group, the results showed that objective bends (150 bp) only emerged at BS1. All results are expressed as the mean ± SD. *p < 0.05 vs. the mock group, ** *p* < 0.01, ***p < 0.001. *n* = 3.

As shown previously in Fig. 4A, the expression of *TFRC* mRNA was remarkably upregulated, and a change in *TFRC* sub-localization to the nucleus was induced by CVB3 (Fig. 4E, L), it inferred that the *TFRC* was activated at the transcriptional level. To verify our hypothesis, we first searched for the *TFRC* promoter region in the UCSC Genome database, and a segment −2000 to +200 bp from the transcription start site (TSS) was identified (Fig. 5A). Then, to determine the function of this region, we generated a plasmid containing the −2000 to +200 fragment fused to a luciferase reporter vector as a TFRCp luciferase reporter gene (Fig. 5B, C). As shown in Fig. 5D, according to a dual-luciferase reporter assay, the activity of the *TFRC* promoter was more than twofold that in the CVB3-infected group compared to the mock group (*p* < 0.001).

To further explore which transcription factors interact with the *TFRC* promoter, we used the JASPAR database (http://jaspar.genereg.net/) for prediction. The *TFRC* promoter region harbor abundant GC boxes. The transcription factor Sp1 was selected for further verification due to its high expression in mRNA levels measured by qPCR in high binding score transcription factors predicted by JASPAR database (Supplementary Fig. 4A). As shown in Fig. 5E, the expression of Sp1 mRNA was increased along with the increase in *TFRC* mRNA expression. The data verified that Sp1 overexpression increased the mRNA and protein levels of *TFRC;* in contrast, Sp1 siRNA decreased *TFRC* mRNA and protein levels (Fig. 5F, G, H). These results demonstrate that Sp1 regulates *TFRC* expression at the transcription level.

To address whether Sp1 affects the promoter activity of *TFRC*, we performed a luciferase reporter assay. Compared to the empty vector, cotransfection with TFRC-Luc and the Sp1 overexpression plasmid resulted in an increase in promoter activity (Fig. 5K), and Sp1 siRNA lowered *TFRC* promoter activity (Fig. 5J). To confirm the exact position of the Sp1-binding site (BS) in the TFRC promoter, we assessed the three GC box motifs (BS1, BS2, BS3, Fig. 5M) using a ChIP assay. The sheared DNA lengths (200–2000 bp) were confirmed by agarose gel electrophoresis (Fig. 5L). As shown in Fig. 5N, O, based on the ChIP- PCR results, in both non-infected and CVB3-infected cells, Sp1 bound to the BS1 region (tgggcatggt) located at −229 to −238 bp but not to BS2 and BS3 on the *TFRC* promoter. These results suggest that Sp1 directly activates *TFRC* transcription by binding to a specific locus on the *TFRC* promoter.

### The protective effect of downregulation of the Sp1/TFRC/Fe axis on ferroptosis

**Fig. 6:**
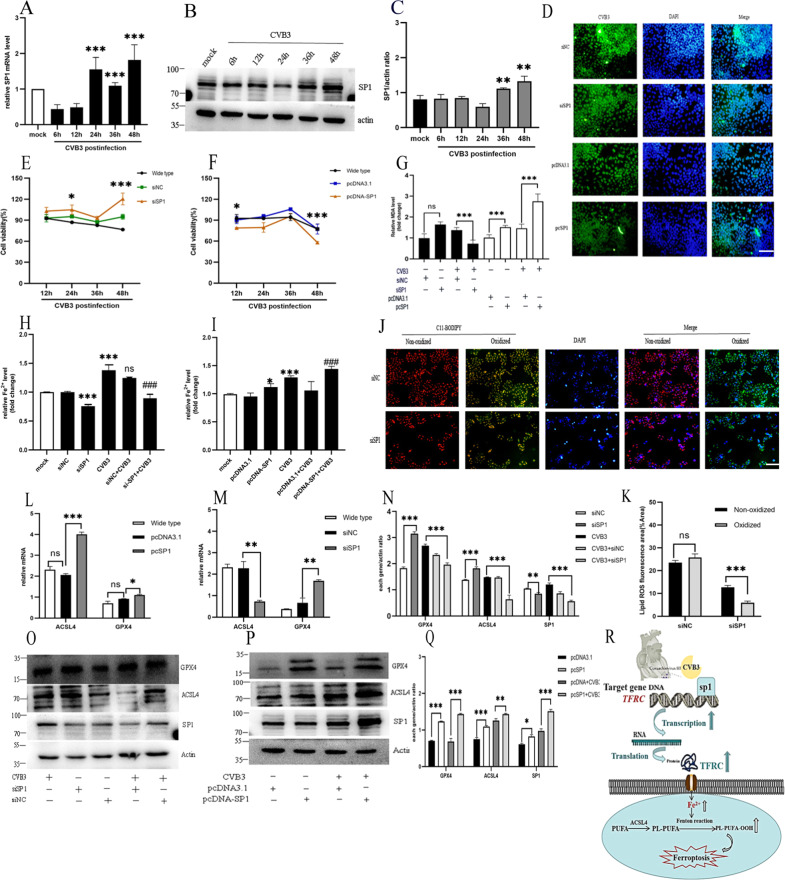
Effect of the transcription factor Sp1 on ferroptosis during CVB3 infection. **A** The mRNA level of Sp1 at different CVB3 infection times was detected via qPCR. **B** The protein level of Sp1 at different CVB3 infection times was recorded via western-blotting. **C** Measurement of the relative gray values of Sp1 in the WB analysis was conducted using ImageJ. **D** CVB3 fluorescence in the SP1 knockdown and overexpression groups was determined via IFA. Cell nuclei were stained with DAPI (blue) (scale bar = 50 μm). **E** Cell viability in the SP1 knockdown group was obviously increased at 48 h postinfection, based on CCK-8 assay results. **F** Cell viability in the Sp1 overexpression group was also decreased at 48 h postinfection. **G** MDA concentrations were determined with Lipid Peroxidation MDA Assay Kit in the Sp1 knockdown and overexpression groups. **H**, **I** Iron concentrations were measured in the SP1 knockdown and overexpression groups using an Iron Assay Kit. **J** The fluorescescence of lipid ROS in the SP1 knockdown group was detected using the BODIPY581/591C11 probe (scale bar = 100 μm). **K** Percentage of oxidized/non-oxidized cells were calculated by ImageJ software according to figure **J**. It suggested that siSP1 could decreased the oxidized lipid ROS significantly (*p* < 0.001). **L**, **M** The effect of Sp1 overexpression and knockdown on ACSL4 and GPX4 mRNA expression was determined by qPCR. **N**, **O** Relative gray value and images of western-blotting in the effect of knockdown Sp1 on ACSL4 and GPX4 protein expression. **P**, **Q** Images of western-blotting and relative gray values in the effect of overexpression Sp1 on ACSL4 and GPX4 protein expression. **R** Schematic illustrating the mechanism by which the Sp1/TFRC/Fe^2+^ cascade facilitates ferroptosis in CVB3-infected HeLa cells. All results are expressed as the mean ± SD. ANOVA was used for comparison between groups. *p < 0.05, **p < 0.01, ***p < 0.001.

Following exposure to CVB3 infection, compared to mock group, the mRNA level of Sp1 increased from 24 h postinfection (Fig. 6A), while the Sp1 protein level increased from 36 h postinfection (Fig. 6B, C). To confirm that CVB3-induced ferroptosis as mediated by Sp1, HeLa cells were transfected with Sp1 siRNA and overexpression vectors and then exposed to CVB3. Similar to the effect of siTFRC, Sp1 downregulation had a protective effect against CVB3-induced ferroptosis, which was reflected by the increase in cell viability (Fig. 6E), reduced MDA levels (Fig. 6G), inhibition of lipid ROS accumulation (Fig. 6J, K) and decreased the iron level (Fig. 6H). Conversely, Sp1 overexpression exhibited a pro-ferroptotic effect, which was evidenced by decreased cell viability (Fig. 6F) and increased levels of MDA (Fig. 6G), elevated cellular iron level (Fig. 6I). Unexpectedly, siSP1 had no obvious reducing effect on virus replication, but in the Sp1 overexpression group, the underlying mechanism deserves further study (Fig. 6D). Notably, we measured the landmark of ferroptosis molecular ACSL4 and GPX4 in the siSP1group at 48 h post CVB3 infection. In line with the effect of siTFR1, Sp1 silencing lowered ACSL4 mRNA and protein levels and increased the GPX4 levels (Fig. 6M, N, O). Conversely, over-expressing increased ACSL4 mRNA and protein compared to NC group (Fig. 6L, P, Q). Taken together, the results demonstrate that transcription factor SP1 is increased in CVB3 infection. It works by increasing the transcription of *TFRC* and associate with ferroptosis. Reduction of SP1 exerting beneficial effects on ferroptosis in CVB3 infection.

In summary, through the Sp1/TFRC/Fe axis (Fig. 6R), CVB3 infection activates the transcription factor Sp1, which targets the *TFRC*, leading to an increase in *TFRC* transcription, enhancement of TFR1 protein expression and an increase in iron pumped into cells. Finally, oxygen-free radicals and lipid peroxide levels are greatly increased, and ferroptosis occurs.

## Discussion

CVB3 is the predominant pathogen of viral myocarditis in children. Viral replication induces direct cell injury including cytoskeletal disruption and triggers uncontrolled inflammation even after viral clearance [24]. Therefore, an effective therapeutic strategy must target both host cells and the virus simultaneously. A soaring number of studies on ferroptosis and metabolism have revealed exciting insights into the molecular and metabolic mechanisms of cell death [25]. Thus, we hypothesized that CVB3 infection may be an important process facilitating the occurrence of ferroptosis. To validate this hypothesis, in this study, we established a CVB3 infection model in vivo and in vitro, measured the levels of ferroptosis markers: cellular iron, lipid peroxide (lipid ROS or MDA), and examined the expression levels of core ferroptosis genes. Notably, in vitro, the time of cell viability decline was consistent with the peak time of virus infection. We found the level of ferrous iron, lipid peroxide were all elevated by CVB3 infection. Acyl-CoA synthetase long-chain family member 4 (ACSL4) and glutathione peroxidase 4(GPX4) are two main mark genes in ferroptosis. ACSL4 dictates ferroptosis sensitivity by shaping cellular lipid composition and is a sensitive pro-ferroptotic gene. The GPX4 utilizes reduced glutathione (GSH) to reduce lipid hydroperoxides to lipid alcohols, protect cells against membrane lipid peroxidation and inhibit ferropotsis [26]. In our study, the pro-ferroptotic genes ACSL4 increased and anti-ferroptotic genes GPX4 decreased (Figs. 1 and 2). These suggested that ferroptosis is activated during CVB3 infection.

Next, we used classical ferroptosis inhibitors ferrostatin-1 (Fer-1) and deferoxamine (DFO), we found the increased iron level, upregulated lipid peroxide and ACSL4 could all be rescued by Fer-1 and DFO in the ferroptosis induced by CVB3 infection. The mechanism by which Fer-1 prevent cell death has been ascribed to its ability to inhibit lipid peroxidation directly by trapping chain-carrying radicals [27]. During the ferroptosis pathway, Fe, could contribute to the ROS pool in the cell through Fenton reaction in which Fe catalyzes the breakdown of H_2_O_2_ to yield hydroxyl radicals. Hence, Fer-1 as a radical-trapping antixodiandts, could inhibit the last step of ferroptosis pathway to slow accumulation of lipid hydroperoxides caused by iron-overload (Fig. 3C, D, F). During the intrinsic pathway of ferroptosis, the expression or activity of intracellular antioxidant enzymes GPX4 is blocked [28]. In our study, we found two ferroptosis inhibitors especially Fer-1 could increase the GPX4 expression, which was consistent with the reports in literature (Fig. 3K, L). Li et al. [29] reported that in diabetic nephropathy mice, iron overload and decreased GPX4 expression were obviously rescued by Fer-1. Liu et al. [30] also found Fer-1 could rescue the downregulated GPX4 in a dose-dependent manner in LPS-induced acute lung injury model in vitro and in vivo. It is mainly dependent on the Fer-1 scavenging of initiating alkoxyl radicals, and the inhibition of the GPX4 activity was relieved. The iron chelator DFO has a significant improvement in life expectancy of thalassemic patients with transfusional iron overload clinically [31]. As results in other experiments [32], we found DFO markedly inhibited increase of ferrous iron, thus rescue the ferroptosis (Fig. 3D).

To further analysis the changes in the metabolites that regulation in ferroptosis with CVB3 infection, we measured the anti-toxisis GSH with infected time points. GSH acts as a regulator of cellular redox state to protect cells from damage caused by lipid peroxides, reactive oxygen and xenobiotics [33]. It is interesting to find that the cell death number was declined at 36 h of infection (Fig. 2C), the lipid peroxide MDA (Fig. 2D), ferrous iron (Fig. 2F), and ACSL4 (Fig. 2H) were all attenuated at 36 h postinfection in accordance with GSH peak level. These indicated that when CVB3 invades and releases harmful substances like lipid peroxide in cellular ferroptosis, host cell initiate GSH as a negative feedback to regulate. However, with imbalance of GSH regulation, the promote ferropotsis factors were increased by CVB3 virus release. At 48 h after CVB3 infection, intracellular free iron reached a peak (Fig. 2F).

As a pivotal indicator and regulator of iron, TFR1 which encoded by TFRC as a critical determinant of ferroptosis sensitivity by modulating cellular iron uptake. TFR1 imports iron from the extracellular environment into cells, contributing to the cellular iron pool required for ferroptosis [34]. In our study, we found TFRC mRNA and protein expression were increased in CVB3 infection model in vivo and in vitro (Figs. 1I, J and 4A, B). To further confirm the function of TFRC in CVB3 infection, we designed siRNA to genetically inhibit TFRC in vitro. TFRC knockdown prevented CVB3-induced ferroptosis in HeLa cells(decreased iron overload, reduced lipid peroxidation, ACSL4 and NCOA4 downregulation, and GPX4 upregulation). Much evidence in reports has shown that TFRC plays an important role in ferroptosis. In an SD heart ischemia/reperfusion (I/R) model, knockdown of TFRC inhibited H/R-induced ferroptosis without p53 deubiquitination [35]. Some researchers have also demonstrated that TFRC-deleted mice exhibit attenuated renal fibrosis, along with reduced renal expression of ferritin and 4-hydroxynonenal, compared with WT mice [36]. During malaria infection, TFRC knockdown decreased parasite-localized lipid peroxides, and led to a substantial decrease in the efficacy of erastin in liver-stage parasite infection [37].

In present study, our team first demonstrated the TFRC was translocation to nuclear with CVB3 infection by IFA and subcellular analysis in western-blot (Fig. 4E, L). Large evidence showed that TFRC can act as a target for infectious diseases because various pathogens use TFRC to enter cells [38, 39]. For example, some New World arenaviruses, which infect humans and cause life-threatening hemorrhagic fever, use TFRC for entry into host cells [40]. Hepatitis C virus also utilizes TFRC for entry in human hepatocytes, and knockdown of TFRC inhibits hepatitis C virus infection in human hepatoma cells [41]. A recent study used erastin to induce lymphoma cell ferroptosis and found that TFRC staining was localized to the plasma membrane and to a perinuclear region associated with the Golgi and the endosomal recycling compartment (ERC) [9]. These all indicate that TFRC could translocation to cytoplasm or nuclear. In our experiment, we further found TFRC recruited to nuclear by a nuclear transcription factor Sp1. Sp1 is a member of the zinc finger family, which bind to specific DNA sequences located in core gene promoter, and interact with the RNA Pol II complex to enhance gene transcription [42]. It is implicated in a variety of essential biological processes, such as cell growth, differentiation, carcinogenesis and apoptosis [43]. In our study, we verified that Sp1 can regulate TFRC transcription and validated that the binding site was located at −229 to −238 bp relative to the TSS using a ChIP assay (Fig. 5D, O). We found that SP1 overexpression upregulated TFRC expression, raised cellular iron levels, increased lipid peroxidation products, and aggravated ferroptosis (Fig. 6F, G, I). Downregulating Sp1 (siSP1) decreased TFRC expression, and rescued the ferroptosis induced by CVB3 infection (Fig. 6E, G, H), which was similar to the effects of siTFRC. This suggests that Sp1/TFRC/Fe forms a functional axis that regulates ferroptosis during CVB3 infection.

## Conclusion

Our study revealed that ferroptosis has an important role in CVB3 infection. Inhibition of ferroptosis by Fer-1, DFO or genetic interference could rescue the cell death and reduce virus replication. Mechanistically, the key iron regulation gene *TFRC* was enhanced by CVB3 infection, leading to the release of large quantities of free iron, which promoted lipid peroxidation and ferroptosis. *TFRC* expression was activated by the transcription factor Sp1, which bound to a specific site in the *TFRC* promoter and increased *TFRC* transcription. Based on these data, we hypothesize that targeting the Sp1/TFRC/Fe pathway may be a potential therapeutic strategy against CVB3-induced injury in the future.

## Supplementary information

supplementary information

Reproducibility checklist

Original western blots data

## Data Availability

The datasets are all presented in main manuscript.
