# Supplementary material for: TFRC upregulation promotes ferroptosis in CVB3 infection via nucleus recruitment of Sp1
Source: Cell Death Dis. 2022 Jul 11;13(7):592. doi: 10.1038/s41419-022-05027-w (PMC9276735; doi:10.1038/s41419-022-05027-w)
Supplement: Supplementary file 1 — supplementary information [file 41419_2022_5027_MOESM1_ESM.pdf]

Supplementary materials

Table 1 Sequences in genes interference

Gene number	Target sequence
genOFFTM st-h-TFRC_001	GTAGGATGGTAACCTCAGA
genOFFTM st-h-TFRC_002	GCACAGCTCTCCTATTGAA
genOFFTM st-h-TFRC_003	GGAGACTTCTTCCGTGCTA
genOFFTM st-h-SP1_001	GCAACATCATTGCTGCTAT
genOFFTM st-h-SP1_002	GCCAATAGCTACTCAACTA
genOFFTM st-h-SP1_003	CTCCCAACTTACAGAACC

Table 2 Primers in experiments in HeLa cells

Primer name	Primer sequence(5'-3')	Tm	Product size
β-actin	F:CATGGAGTCCTGTGGCATC	59℃	157bp
	R:CAGGGCAGTGATCTCCTTCT		
ACSL4	F:AATGCAGCCAAATGGAAAAG	60℃	152bp
	R:CACAGAAGATGGCAATGGTG		
GPX4	F:CAGTGAGGCAAGACCGAAGT	59℃	111bp
	R:CTGCTTCCCGAACTGGTTAC		
NCOA4	F:GCACTTGATGGCTCATGCTA	60℃	151bp
	R:ATAACCACTGGCAGGTTTGC		
Fth1	F:TGACAAAAATGACCCCCATT	59℃	160bp
	R:CAGGGTGTGCTTGTCAAAGA		
TFRC	F:AAAATCCGGTGTAGGCACAG	59℃	179bp
	R:TTAAATGCAGGGACGAAAGG		
SP1	F:TCATACCAGGTGCAAACCAA	60℃	224bp
	R:GCTGGGAGTCAAGGTAGCTG		
MAVS	F:CCTAAGGCCCTCTCTTTGCT	59℃	185bp
	R:GCACCTCCAAAGAGCTTGAC		

TFAP2A	F:ACTGAGACTCCCGTCAATGG	60℃	231bp
	R:GCGTGTTCCTTAATCCGTGT		
STAT3	F:CTGGCCTTTGGTGTTGAAAT	59℃	202bp
	R:AAGGCACCCACAGAAACAAC		
TFRCp-BS1	F:GAGCCCAGGAGTTCAAGACTA	58℃	150bp
	R:ATTCCTGACCTCAGGTGATCT		
TFRCp-BS2	F:TACGTGCCTCAGGAAGTGAC	58℃	184bp
	R:AGTGGCAGAAACAGTGGATG		
TFRCp-BS3	F:GTACGTGCCTCAGGAAGTGA	58℃	150bp
	R:GAAATGACAACGAGGGGATG		

Table 3 Primers in experiments in Mice

Primer name	Primer sequence(5'-3')	Tm	Product size
β-actin	F: GCTACAGCTTCACCACCACA	58℃	208bp
	R:AAGGAAGGCTGGAAAAGAGC		
CVB3	F:GGCGCTAGCACTCTGGTATC	59℃	191bp
	R:CGAACGCTTTCTCCTTCAAC		

Table Legends

Table 1 The sequences of small interferring RNA (siRNA) oligonucleotide of Human. Each gene (TFRC and SP1) were designed with three interfering target sequences which verified by q-PCR and Western-blot.

Table 2 The primer of gene's sequence, annealing temperature and product size in PCR in HeLa cells.

Table 3 The primer of gene's sequence, annealing temperature and product size in PCR in mice heart tissue.

Supplementary Figures

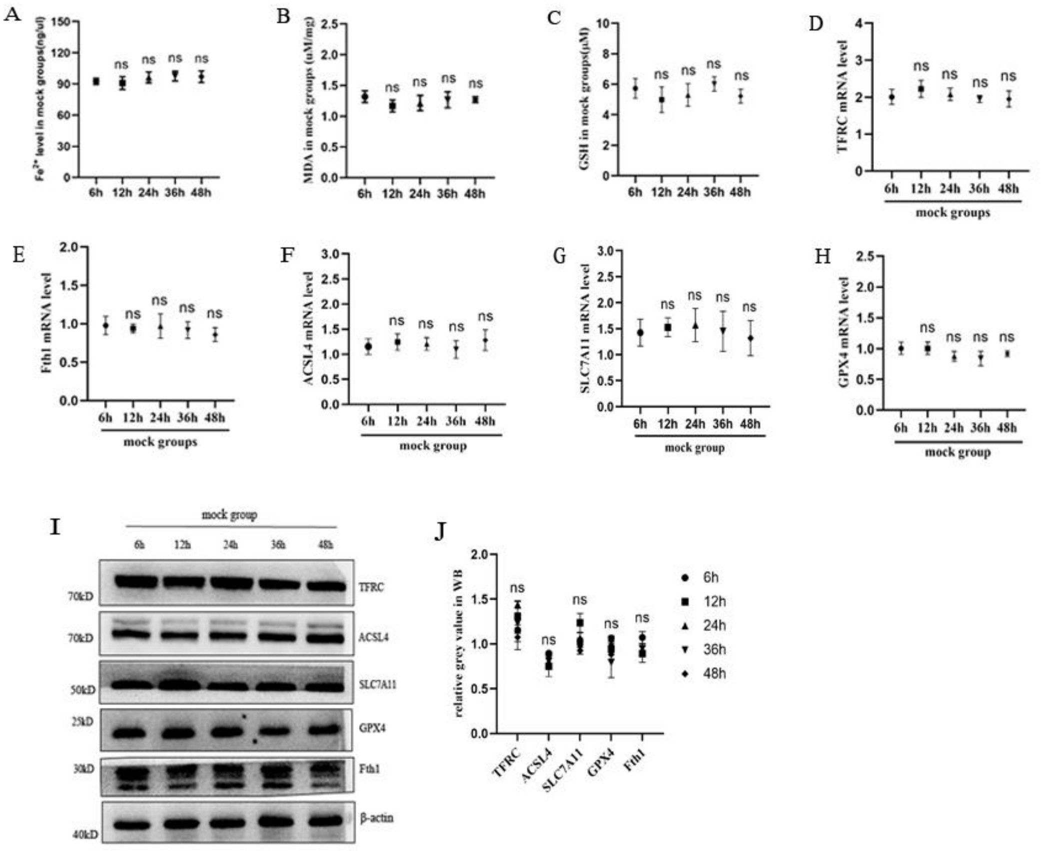

Supplement Fig. 1 The characteristics of ferroptosis measured at each time point of CVB3 infection in normal control group (mock group) of HeLa cell. **(a)** Iron concentrations were tested in the each time point mock groups using an Iron Assay Kit. **(b)** MDA concentrations were determined with Lipid Peroxidation MDA Assay Kit in the different mock groups. **(c)** GSH in all time point mock groups of HeLa cells were detected using the GSH assay kit. **(d-h)** The mRNA levels of ferroptosis genes were detected via qPCR at each time point of mock group in HeLa cell. **(i)** Images of Western-blotting of ferroptosis gene protein expressions at each time point of mock group in HeLa cell. **(j)** Relative gray values of Western-blotting. All results are expressed as the mean ±SD. T-test was used for pairwise comparison. ANOVA was used for comparison between groups. ns $p > 0.05$ vs. the group of 6 hours, *$p < 0.05$, ** $p < 0.01$, ***$p < 0.001$. n=3.

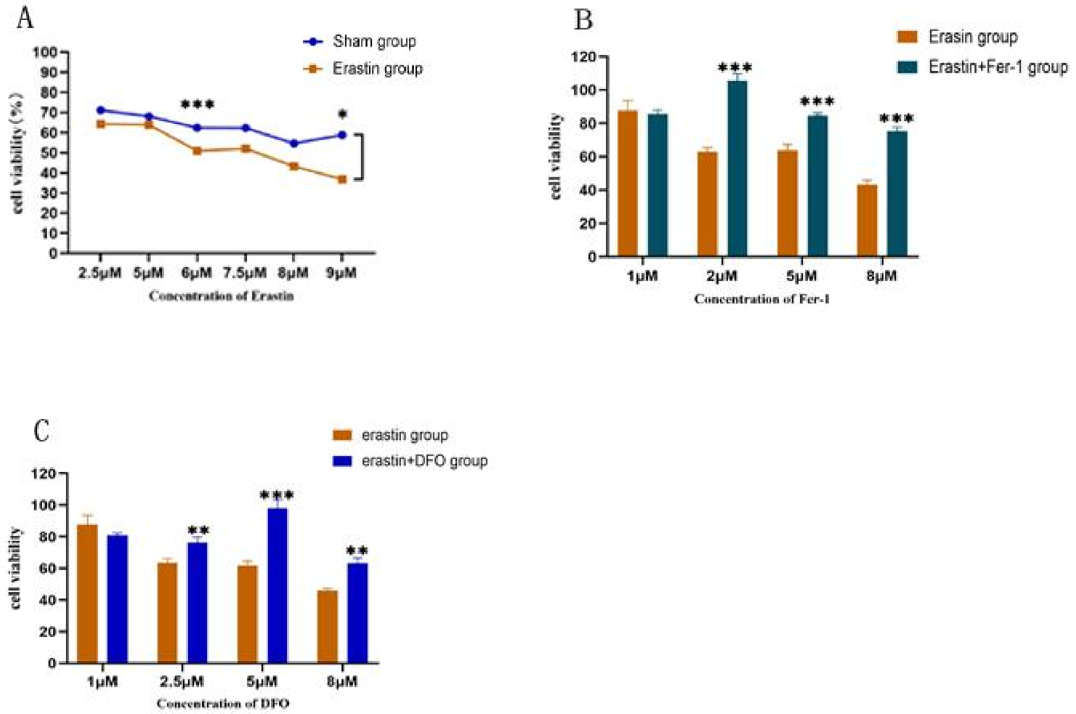

Supplement Fig. 2 The effect of ferroptosis inducer and inhibitors on the cell viability of HeLa cell. **(a)** The cellThe effect of ferroptosis inducer and inhibitors on the cell viability of HeLa cell. viability of HeLa intervened with ferroptosis inducer Erastin at various concentrations. It indicated that the optimum concentration of Erastin on HeLa was 6 μM. **(b)** The effect of different concentrations of inhibitor ferrostatin-1 (Fer-1) on rescue HeLa cell death induced by Erastin. It showed that the minimum effective concentration of Fer-1 on Erastin is 2 μM. **(c)** The effect of ferroptosis inhibitor deferoxamine mesylate (DFO) on rescue HeLa cell death induced by Erastin. It showed that the most effective concentration of DFO on Erastin is 5 μM. T-test was used for pairwise comparison. *$p < 0.05$, ** $p < 0.01$, ***$p < 0.001$. n=6.

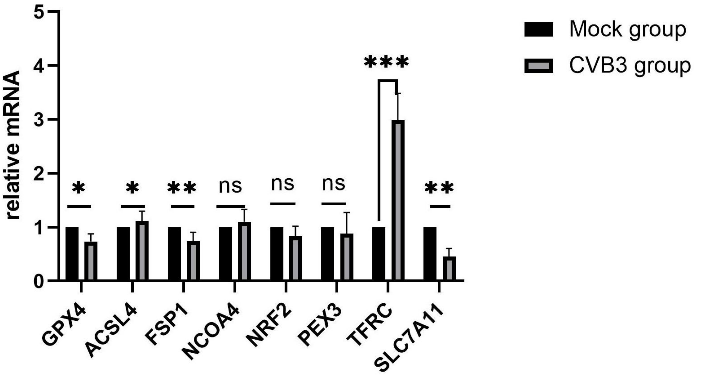

Supplement Fig. 3 The mRNA levels of ferroptosis related genes were detected via qPCR on mock group and CVB3 group of HeLa cells. It indicated that TFRC was increased obviously after CVB3 infection compared to mock group. T-test was used for pairwise comparison. ns $p > 0.05$, *$p < 0.05$, ** $p < 0.01$, ***$p < 0.001$. n=6.

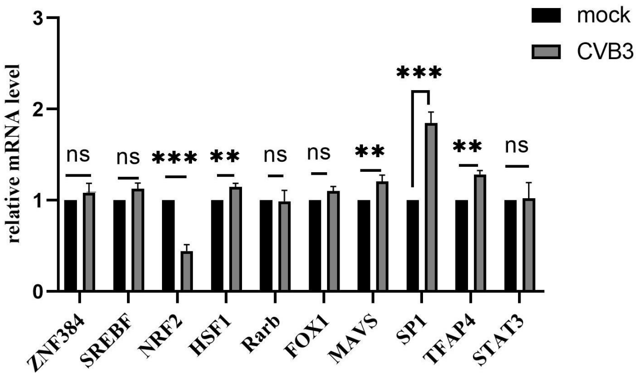

Supplement Fig. 4 The mRNA levels of top 10 transcription factors act on TFRC predicted by JASPAR were detected via qPCR. It indicated that transcription factor SP1 was increased significantly most after CVB3 infection compared to mock group. T-test was used for pairwise comparison. ns $p > 0.05$, *$p < 0.05$, ** $p < 0.01$, ***$p < 0.001$. n=6.
