# Supplementary figures and images for: TFRC upregulation promotes ferroptosis in CVB3 infection via nucleus recruitment of Sp1
Source: Cell Death Dis. 2022 Jul 11;13(7):592. doi: 10.1038/s41419-022-05027-w (PMC9276735; doi:10.1038/s41419-022-05027-w)

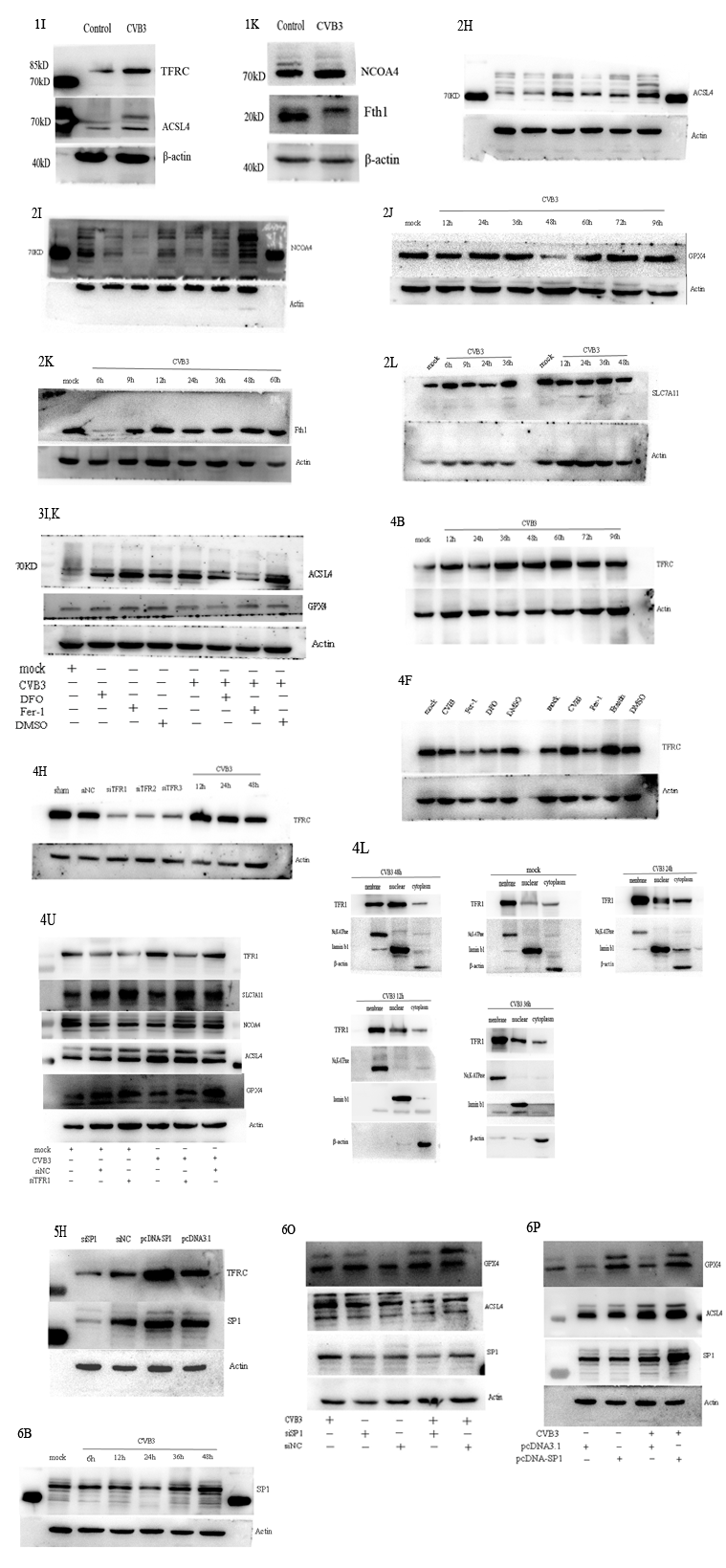

Supplement: Supplementary file 3 — Original western blots data [file 41419_2022_5027_MOESM3_ESM.tif]
